# Networking and Specificity-Changing DNA Methyltransferases in *Helicobacter pylori*

**DOI:** 10.3389/fmicb.2020.01628

**Published:** 2020-07-17

**Authors:** Hirokazu Yano, Md. Zobaidul Alam, Emiko Rimbara, Tomoko F. Shibata, Masaki Fukuyo, Yoshikazu Furuta, Tomoaki Nishiyama, Shuji Shigenobu, Mitsuyasu Hasebe, Atsushi Toyoda, Yutaka Suzuki, Sumio Sugano, Keigo Shibayama, Ichizo Kobayashi

**Affiliations:** ^1^Department of Computational Biology and Medical Sciences, Graduate School of Frontier Sciences, The University of Tokyo, Tokyo, Japan; ^2^Institute of Medical Science, The University of Tokyo, Tokyo, Japan; ^3^Department of Bacteriology II, National Institute of Infectious Diseases (NIID), Musashimurayama, Japan; ^4^National Institute for Basic Biology (NIBB), Okazaki, Japan; ^5^School of Medicine, Chiba University, Chiba, Japan; ^6^Advanced Science Research Center, Kanazawa University, Kanazawa, Japan; ^7^Department of Basic Biology, School of Life Sciences, SOKENDAI (The Graduate University for Advanced Studies), Okazaki, Japan; ^8^Advanced Genomics Center, National Institute of Genetics, Mishima, Japan; ^9^Department of Infectious Diseases, School of Medicine, Kyorin University, Mitaka, Japan; ^10^Institut de Biologie Intégrative de la Cellule (I2BC), Université Paris-Saclay, Gif-sur-Yvette, France; ^11^Research Center for Micro-Nano Technology, Hosei University, Koganei, Japan

**Keywords:** epigenetics, methylome, DNA methylation, gastric cancer, epigenome, DNA methyltransferase, SMRT, Pacbio

## Abstract

Epigenetic DNA base methylation plays important roles in gene expression regulation. We here describe a gene expression regulation network consisting of many DNA methyltransferases each frequently changing its target sequence-specificity. Our object *Helicobacter pylori*, a bacterium responsible for most incidence of stomach cancer, carries a large and variable repertoire of sequence-specific DNA methyltransferases. By creating a dozen of single-gene knockout strains for the methyltransferases, we revealed that they form a network controlling methylome, transcriptome and adaptive phenotype sets. The methyltransferases interact with each other in a hierarchical way, sometimes regulated positively by one methyltransferase but negatively with another. Motility, oxidative stress tolerance and DNA damage repair are likewise regulated by multiple methyltransferases. Their regulation sometimes involves translation start and stop codons suggesting coupling of methylation, transcription and translation. The methyltransferases frequently change their sequence-specificity through gene conversion of their target recognition domain and switch their target sets to remodel the network. The emerging picture of a metamorphosing gene regulation network, or *firework*, consisting of epigenetic systems ever-changing their specificity in search for adaptation, provides a new paradigm in understanding global gene regulation and adaptive evolution.

## Introduction

**FIGURE 1:**
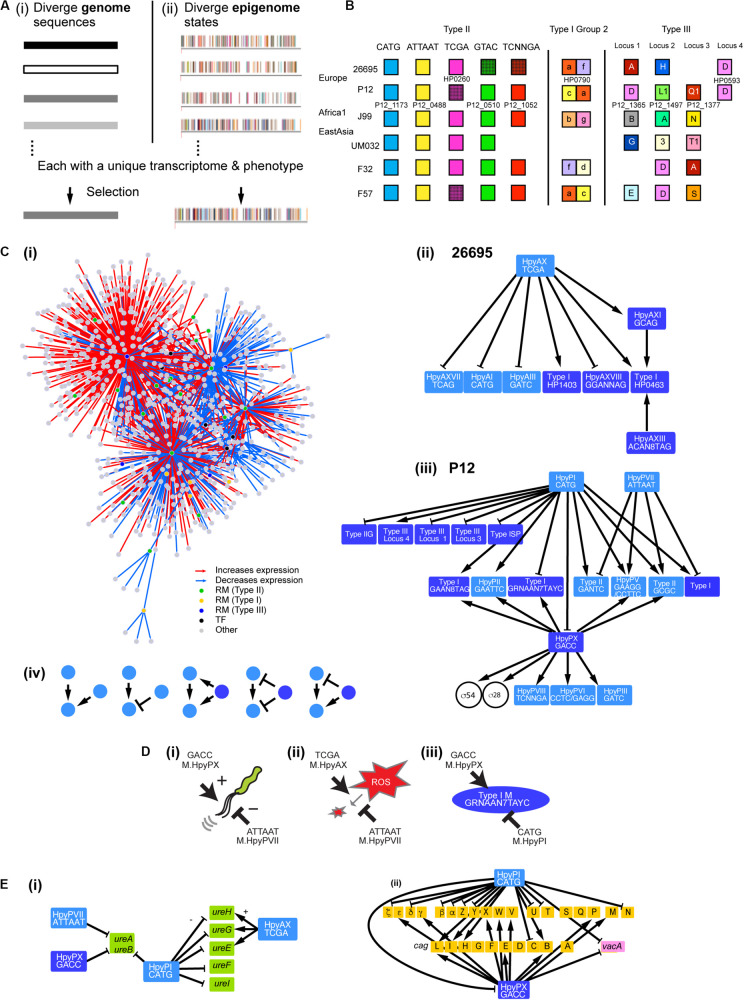
Gene regulation network involving multiple DNA methyltransferases. **(A)** Two models for adaptive evolution. **(i)** Genome-based. **(ii)** Epigenome-based model with a part of the methylomes (*rpoB*, top strand) of closely related *H. pylori* strains [modified from Furuta et al. (2014)]. One bar indicates one methylation and one color indicates one methylation sequence motif. **(B)** Homology grouping of Target Recognition Domains in the analyzed restriction-modification loci in some global strains (Lin et al., 2001; Furuta et al., 2011; Furuta and Kobayashi, 2012b; Kojima et al., 2016). **(C) (i)** Gene regulation network in *H. pylori* deduced from this work. It shows hub DNA methyltransferases of various types, other transcription factors and their target genes. Interaction of DNA methyltransferases **(ii)** in strain 26695 and **(iii)** in strain P12. An arrow indicates a positive effect while a T shape indicates a negative effect. Type II RM systems including solitary DNA methyltransferases are shown in a light blue box. Type I RM systems and Type III RM systems, in which TRD movement causes allelic variation, are shown in a dark blue box. Two transcription factors (sigma factors) are shown in a circle. **(iv)** Five elementary patterns of interaction between DNA methyltransferases identified in the network. **(D)** Opposite roles of two methylation systems in regulation. **(i)** Swimming motility. **(ii)** ROS metabolism. **(iii)** Methyltransferase expression. A DNA sequence indicates a methylation motif. **(E)** Regulation of virulence-related genes. **(i)** Genes associated with urease synthesis and maturation. **(ii)** Genes in the *cag* pathogenicity island (brown) and *vacA* gene. Light blue, Type II methyltransferase; dark blue, Type III methyltransferase.

The currently dominating model for adaptive evolution assuming selection from diverse genome sequences is derived from genetics and molecular biology of microorganisms and other forms of life, but its general validity became thoroughly testable only recently with the availability of many genome sequences within a species. Meanwhile studies of cell differentiation in multicellular organisms have revealed critical roles of epigenetics, here defined as information added to genome nucleotide sequences and heritable through genome replication. The diverse epigenomes, as opposed to the diverse genomes, may be regarded as the units of evolution (Figure 1A). There are indeed increasing lines of evidence for *trans-*generation epigenetic inheritance in plants and animals (Miska and Ferguson-Smith, 2016; Quadrana and Colot, 2016). In unicellular and equally dividing bacteria, a somatic cell can be regarded as a germ line cell, to the first approximation, which simplifies the issue. Sequence-specific DNA base methylation there affects gene expression (Sánchez-Romero et al., 2015) and its reversible changes can lead to different phenotype sets (De Ste Croix et al., 2017; Gorrell and Kwok, 2017). A DNA methyltransferase (MTase) is often paired with a restriction enzyme to form a restriction-modification (RM) system (Roberts et al., 2003). They form a prokaryotic immune system attacking non-self DNA lacking proper epigenetic DNA methylation and also behave as selfish mobile elements (Vasu and Nagaraja, 2013).

Prokaryotes possess, on average, five DNA methyltransferase genes and three methylated sequence motifs (Blow et al., 2016; REBASE PacBio statistics, 2020). A large repertoire of DNA methylation systems, with twenty to thirty genes per genome, has been found in *Helicobacter pylori* (Furuta et al., 2014; Krebes et al., 2014). They colonize on children’s stomach cells using their motility, persist there for decades, tolerating attacks with ROS (reactive oxygen species) by immune systems and repairing DNA damages, and may eventually cause ulcer and cancer (Backert and Yamaoka, 2016). Some of their RM systems are conserved, while some others are present only in few lineages (Figure 1B; Vale et al., 2009; Uchiyama et al., 2016). Many are obtained through horizontal transfer, within the species or from a different species, and decay by mutation (Lin et al., 2001; Furuta et al., 2011; Furuta and Kobayashi, 2012b). In some RM types (Type I, Type III, Type IIG), target sequence recognition domains (TRDs) move within a gene and between genes, sometimes from various (eu)bacterial species beyond phylogenetic barriers (Furuta et al., 2011; Furuta and Kobayashi, 2012b). Because of these processes, the repertoire of methylation systems changes rapidly and apparently irreversibly during lineage diversification of *H. pylori* (Kojima et al., 2016). Single-molecule real-time sequencing technology indeed revealed highly diverged methylomes even for closely related genomes (Furuta et al., 2014). Each of the methylomes may have a unique gene expression pattern and phenotype set and might be regarded as the units of adaptive evolution (Figure 1A; Furuta and Kobayashi, 2012a). Previous experiments have indeed found effects of individual DNA methylation genes on the transcriptome and phenotype in many bacterial species (Srikhanta et al., 2009, 2011; Fang et al., 2012; Kumar et al., 2012; Furuta et al., 2014; Sánchez-Romero et al., 2015; Zhang et al., 2017; Nye et al., 2019).

In order to examine the above *epigenetics-based adaptive evolution* model, we here knocked out ten of specificity-determinant genes of DNA methylation systems, examined the resulting methylomes and transcriptomes, and predicted and tested adaptation-related phenotype changes. We focused on Type III and Type I RM systems, which frequently change sequence specificity by domain movement (Furuta et al., 2011, 2014; Furuta and Kobayashi, 2012b), and several uncharacterized Type II RM systems.

Our results revealed a huge gene regulation network that involves many, interacting and ever-changing DNA methylation systems as hub transcription factors (Figure 1C). It controls gene expression and adaptive phenotype sets in a unique way.

## Materials and Methods

### Bacterial Strains, Culture Media and Plasmids

The strains, plasmids and their relevant characteristics are shown in Supplementary Table S3 of the supporting information. *E. coli* strain was cultured using Luria-Bertini (LB, Lennox) broth (10 g tryptone, 5 g Yeast extract, 5 g NaCl per 1L). Solid medium (LB-agar) was prepared by addition of 1.5% agar [Nakalai tesque, Kyoto Japan]. If necessary, kanamycin (Km) and chloramphenicol (Cm) [FUJIFILM Wako Pure Chemical Corporation, Osaka, Japan] was added to LB or LB-agar at 50 and 25 μg/ml, respectively. Brucella Broth (BB) [BD Bioscience, San Jose, CA, United States] supplemented with 10% fetal bovine serum (BB-FBS) [Cell Culture Laboratories, Cincinnati, OH, United States] was used for liquid culture of *H. pylori*. The solid media used were BB-FBS agar (BB-FBS, 1.5% agar), BB-HB agar (BB supplemented with 5% horse blood, 1.5% agar), or BD BBL Trypticase Soy Agar with 5% Sheep Blood (TSA) [BD Bioscience]. If necessary, Km and Cm were added to BB-FBS agar at 15 and 5 μg/ml, respectively. Both liquid cultures and agar plates were incubated at 37°C in the presence of 10% CO_2_ and 5% O_2_.

### DNA Manipulation and Mutant Construction

The oligonucleotides used in this study are listed in Supplementary Table S4. KOD FX neo (Toyobo, Ohtsu, Japan) was used as the DNA polymerase for PCR. *E. coli* HST08 chemical competent cells (Takara Bio, Kusatsu, Japan) and In-Fusion HD cloning kit (Takara Bio, Kusatsu, Japan) were used for conventional cloning. Gene-Elute^TM^ plasmid DNA kit (Sigma-Aldrich, St. Louis, MO, United States) was used for plasmid purification.

For gene knockout experiments, a region of approximately 800 bp flanking an MTase gene or the specificity subunit gene was cloned into pUC119 or pUC19 together with a fragment containing a Km resistance gene (*aphA*) with a promoter derived from pHel3 (Heuermann and Haas, 1998). The resulting constructs (Supplementary Table S3) were transferred to competent cells prepared in 300 μM sucrose by electroporation, and the transformants were selected on BB-FBS agar containing Km. After two rounds of single colony isolation, the clones were stocked. The absence of the original locus from these mutants was confirmed using PCR. To disrupt methyltransferase gene of a Type II RM system, R gene alone was replaced with the Cm gene first. Then, in the second recombination experiment, the Cm gene and the M gene were replaced together by the Km gene.

In gene restoration experiments, the original methyltransferase gene (*hpyAXM, hpyPVIIM*) was PCR-amplified from genomic DNA and joined with Cm resistance gene on pUC19 with their respective c.a. 800-bp upstream and downstream fragments. In parallel, a construct without the MTase gene was also made. These constructs were transferred into strain PIK14 (Δ*hpyAXM*) or PIK64 (Δ*hpyPVIIM*) (Supplementary Figure S4). The absence of the initially integrated Km allele from these mutants was confirmed by PCR and sequencing.

### Methylome Decoding

Total DNA was extracted from a stationary-phase liquid culture of each *H. pylori* strain, PIK38, PIK39, PIK40, by lysozyme treatment and phenol-chloroform extraction. The DNA was further purified using Qiagen Genomic-tip 100-G column (Qiagen, Hilden, Germany) following the manufacture’s protocol. The DNA was sheared to ∼20 kb using a g-Tube (Covaris, Woburn, MA, United States) and the libraries for sequencing were constructed with SMRTbell Template Prep Kit 1.0 (Pacific Biosciences of California, Menlo Park, CA, United States) following the standard instruction for 20-kb template preparation. In the process of size selection using BluePippin (Sage Science, Beverly, MA, United States), 10–50 kb molecules were collected with high-pass v3 program. Sequencing was performed using PacBio RS II (Pacific Biosciences of California, Menlo Park, CA, United States) with P5-C3 chemistry and 3-h movie. Data assembly was performed using the program HGAP (Chin et al., 2013) packaged in SMRT Analysis v2.2.0.

The re-sequenced strain P12 genome was annotated by MiGAP pipeline in National Institute of Genetics, Japan (Sugawara et al., 2009), and then the updated annotation was used for RNA-seq data analysis.

Methylomes of strain 26695, PIK14, PIK16, and PIK17 were decoded to confirm the loss of methylation at the expected sites. For this analysis, we used stationary phase cell culture and Genomic-tip 100-G column.

### Transcriptome Analysis

Total RNA was extracted from *H. pylori* liquid culture at exponential phase (OD_600 nm_ = 0.40–0.45 in a 1-cm path cuvette) using a Pure-Link RNA mini kit (Lifetechnologies, Carlsbad, CA, United States). The rRNA was depleted from the total RNA using a RiboZero gram-negative kit (Illumina, San Diego, CA, United States) prior to cDNA synthesis. cDNA libraries of 200 to 400-bp fragment size were constructed using a TruSeq mRNA kit (Illumina, San Diego, CA, United States) for strain 26695 and its derivatives, or SureSelect Strand Specific RNA library kit (Agilent Technologies, Santa Clara, CA, United States) for P12 and its derivatives. Indexed cDNA libraries were pooled and sequenced in paired-end mode under a HiSeq 2500 platform with unrelated samples.

Reads of 100 bp were mapped on to the strain 26695 genome (RefseqID: NC_000915.1) or P12 genome (our re-sequenced data) using the BWA program, allowing 4% mismatches. This gave rise to 17 to 31 million mapped reads per sample for 26695 and its derivative samples, and 6.5 to 27 million mapped reads for P12 and its derivative samples. The counts of mapped reads per gene were obtained using HTSeq 0.6 (Anders et al., 2015). The read depth was calculated using BEDtools (Quinlan and Hall, 2010). Differential gene expression analysis was conducted for two sets of biological replicate data using Bioconductor package TCC (Sun et al., 2013). In TCC, we used a TMM normalization-edgeR iteration protocol. In this work, the genes with *q*.value (false discovery rate) < 0.01 and *a*.value (mean expression value in log_2_) > 0.75 were considered to be differentially expressed genes (DEGs).

Pathway activity analysis (Lee et al., 2008) was performed using Bioconductor package GSVA (Hänzelmann et al., 2013). TMM-normalized read count data was used as input. 71 KEGG pathway categories that have at least five gene members in *H. pylori* genome were considered. Gene expression changes in members of representative KEGG categories were visualized using Pathview (Luo and Brouwer, 2013).

For stain P12, transcription unit data was not available. Therefore, we tentatively, defined regulatory regions of each coding sequence as start codon plus 149 bp upstream region (152 bp in total) for each coding sequence. Sequence motifs search for the genomic segments and sequence retrieval were conducted using standard functions implemented in R package Biostrings (Pages et al., 2003). We used Marlov Maximal order model as the number of expected motif count (Rocha et al., 1998). Motif frequency (Mr) was defined as the ratio of observed counts to expected counts.

### qPCR

The qPCR was performed for validation of the transcriptome data (Supplementary Table S5). This was performed for total RNA before rRNA depletion, using a StepOnePlus real-time PCR system (Life Technologies), ReveTra Ace RT-PCR (Toyobo), and KOD SYBR qPCR kit (Toyobo) by the absolute quantitation method described in the manufacturers’ protocols. Total RNA was first converted to cDNA, and then used as a qPCR template. A reference gene, which showed similar levels of expression in all the strains in the RNA-seq experiments, was used as a reference copy number for cDNA. This was HP1035 for 26695 and its derivatives’ samples, *lepB* (HPP12_0582) for strain P12 and its *mod* gene KO samples, *purD* (HPP12_1183) for *hpyPVIIM* KO and paired P12 samples, and *horF* (HPP12_0684) for *hpyPIM* KO and paired P12 samples, respectively. The quantity of cDNA was determined on the basis of standard curves with *R*^2^ > 0.99. The qPCR primers used are listed in Supplementary Table S4.

### Growth

From the 50% glycerol stock, each strain was streaked on four TSA plates and incubated for 2 days. The colonies of one plate was pooled in 1 ml BB and then transferred to a Petri dish containing 10 ml BB-FBS and incubated at 37°C in the presence of 10% CO_2_ and 5% O_2_ in a CO_2_ incubator for 24 h. After incubation, the liquid culture was diluted to OD_600 nm_ = 0.1 in 30 ml fresh BB medium with 10% FBS and incubated in the CO_2_ incubator with agitation (rpm-90) for 4 days, and OD_600 nm_ was recorded at 6, 12, 24, 30, 48, and 72 h (or 6, 12, 24, 36, and 48 h for strain 26695 data set) after inoculation. At the same time, serial dilution of each strain was made and spread on BB-HB agar. After 3 days of incubation, colonies were counted and cfu (colony forming unit) was calculated.

### Oxidative Stress Resistance Test

Liquid culture was prepared in triplicate for each strain described above. They were then diluted in BB to OD_600 nm_ = 0.8. After dilution, 100 μl of the cell suspension was spread onto BB-HB agar with a cotton swab. A sterile paper disk (5 mm in diameter) was placed on the center of the plate. To assay peroxide sensitivity, 7.5 μl of a reactive oxygen species (ROS)-inducing agent [30% hydrogen peroxide (HP) (Wako Pure Chemical Ind. Ltd.) or 5% *tert*-butyl hydroperoxide (Wako Pure Chemical Ind. Ltd.) was placed on the center of the disk. After 3 days of incubation at 37°C in the CO_2_ incubator as described above, the diameter of the zone of inhibition (mm) was measured. The diameter of the disk was subtracted. To evaluate the significance for the difference between the control and the mutant, we used two-tailed Student’s *t*-test or Welch *t*-test after testing equality of variance by *F*-test assuming normal distribution as we could have only small sample size in each experiment.

### Mitomycin C Sensitivity Test

Liquid cultures in triplicate were prepared for each strain as described above and then diluted in BB to OD_600 nm_ = 0.8. After dilution, 100 μl of the cell suspension was spread onto BB-HB agar using a cotton swab. A sterile paper disk (5 mm in diameter) was placed on the center of the plate. To assay sensitivity against DNA damaging agent, 7.5 μl of mitomycin C (10 μg/ml) (Sigma-Aldrich, St. Louis, MO, United States) was placed on the center of the disk. After 3 days of incubation at 37°C in the CO_2_ incubator, the diameter of the zone of inhibition (mm) was measured as described above. The diameter of the disk was subtracted. The significance of the difference was evaluated using *t*-test as above.

### Motility Assay

From the stock, each strain was streaked on three TSA plates and incubated Luria for 2 overnights Luria After incubation, 1 ml BB Luria was spread on the colonies and then they were scraped off with a sterile loop. Then 200 μl of the liquid culture was mixed with 10 ml BB-FBS Luria and incubated overnight at 70 rpm. Next day, the culture was diluted to OD_600_ of 0.8 and 5 μl of the culture was spotted on the center of BB-FBS soft agar [0.35% agar (w/v)]. Optionally, 50 μg ml^–1^ triphenyl tetrazolium chloride (Sigma-Aldrich, St. Louis, MO, United Kingdom) was added to visualize pH shift around the cells. After 5 days of incubation, the diameter of bacterial swimming zone with central inoculation spot was measured. The significance of the difference was evaluated using *t*-test as described above.

## Results

### Diverse DNA Methyltransferases and Their Target Motifs Shaping the Methylome

**FIGURE 2:**
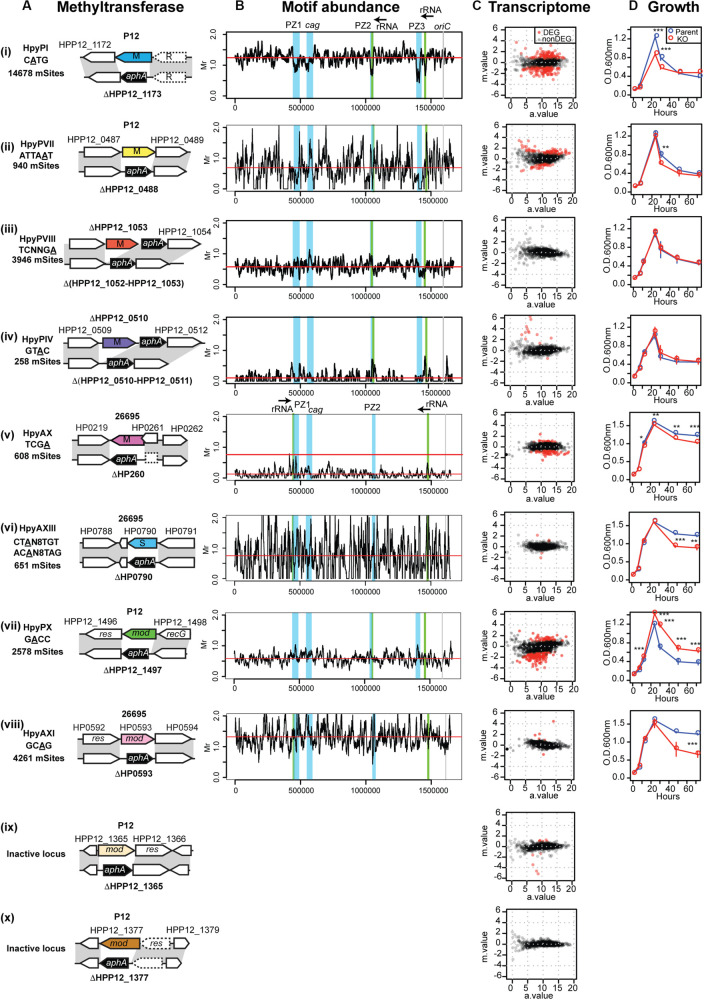
Methyltransferases, their target distribution, knockouts’ effects on transcriptome and growth. **(A)** Methlyltransferase name, target motif with methylated base underlined, and its number per genome (mSites). Parental strain (P12 or 26695), locus tag, genetic maps around the methylation specificity gene and its knockout. **(B)** Different distribution of relative abundance of methylation motifs along the genome. The four major genomic islands are highlighted in blue. Motif frequency was determined for a 10-kb overlapping sliding window. Motif frequency (Mr) was defined as the ratio of the observed count to the count expected from a Markov model. Red horizontal bar indicates the median. **(C)** Transcriptome response to DNA methyltransferase gene knockout, shown as a MA plot with *X*-axis for average read count across samples (*a*.value) and *Y*-axis for fold change in log2 (knockout mutant/parent [*m*.value)]. Red dot indicates differentially expressed genes (DEGs) (*q*.value < 0.01, *m*.value ≥ 0.75). The transcriptome data of HpyPX (GTAC) knockout of P12 **(C (iv))** was published earlier (Zhang et al., 2017). **(D)** Culture OD during 4-day incubation period. A point and an error bar indicate the mean and the SEM, respectively.

We knocked out each of the specificity-determinant genes of a total of 10 known/putative DNA methylation systems (Figure 2A) in two laboratory strains, P12 and 26695. Although both belong to hpEurope population (Backert and Yamaoka, 2016), they differ in the repertoire of RM systems (Figure 1B). RM systems are classified into three (Roberts et al., 2003). Type I RM systems are composed of three subunits: a restriction (R) enzyme subunit, a methyltransferase (M) subunit, and a specificity (S) subunit usually with two target recognition domains (TRDs). In Type II RM systems, R enzyme and M enzyme separately recognize a sequence and M enzyme methylates both strands. In Type IIP RM systems among them, the recognition sequence is a palindrome. Type III RM systems are likewise composed of R and M proteins, but their TRD is present within the M protein and methylation takes place on one strand of a non-palindromic target sequence.

By decoding methylome of P12 and the knockouts, we detected modification of A or C in 15 motifs (Supplementary Table S1) [see also (Furuta et al., 2014)]. A methylation occurred at 2,578/2,579 sites of the GACC motif in the chromosome, but its methylation was very much decreased in ΔHPP12_1497 (Supplementary Table S1). We concluded that GACC is the recognition motif of the HPP12_1497 product for A methylation and propose to designate this Type III RM system as HpyPX. The other two Type III RM loci in P12 examined (Figure 2A) are not related to any methylation activity detected (Supplementary Table S1). Methylation activity of locus 3 was not detected in the other strains examined (Furuta et al., 2014; Krebes et al., 2014; Lee et al., 2015). As this locus is conserved and fosters allelic TRD variation (Figure 1B; Furuta and Kobayashi, 2012b; Kojima et al., 2016), it may have some unknown function. We further created P12 mutants knocked out for three Type II MTase genes (HpyPI, HpyPVII, HpyPVIII) and 26695 mutants for a Type II MTase gene (HpyAX), a Type III MTase gene (HpyAXI), and a Type I specificity gene (HpyAXIII) (Figures 1B, 2A). The 26695 knockout mutants lost methylation at the expected motifs (Krebes et al., 2014): HpyAX, TCGA; HpyAXIII, CTAN_8_TGT and ACAN_8_TAG; HpyAXI, GCAG (Supplementary Table S1).

An earlier work examined the distribution of methylation motifs along the genome (Furuta et al., 2014). The genomic islands referred to as plasticity zones (Kersulyte et al., 2009), or Integrative Conjugative Elements (Fischer et al., 2010), apparently avoid occurrence of m4C, and m6A methylation (Furuta et al., 2014). We now analyzed the distribution of each of the above methylation motifs in comparison with the expected frequency (Figure 2B). Their average frequency turned out to vary greatly with GTAC and TCGA strongly avoided (Humbert and Salama, 2008; Zhang et al., 2017). The CATG motif of a conserved Type IIP RM system turned out to occur less in the genomic islands including cagPAI (cag pathogenicity island) (Supplementary Figure S1, left), but this is not the case for the other motifs (Figure 2B). For GACC motif in particular, the sliding windows overlapping with PZ1 and PZ3 showed the highest abundance and those overlapping with PZ2 and the neighboring 23 rRNA gene showed a relatively high abundance (Supplementary Figure S1 right, Supplementary Table S2).

### Gene Regulation Network Involving Many Sequence-Specific MTases as Hub Regulators

Gene regulation network of *H. pylori* was proposed to be built shallow with only few transcription factors (Danielli et al., 2010). However, our transcriptome analysis of the MTase knockouts (Figure 2C, Supplementary Data Set S1) revealed a large network involving many MTases as hub regulators (Figure 1C). The MTases affect expression of virulence factors such as urease-related genes, cagPAI genes and *vacA* among others (Figure 1E). The MTases varied widely in their effects on the transcriptome (Figure 2C). MTases for CATG (M.HpyPI), ATTAAT (M.HpyPVII), GACC (M.HpyPIX), TCGA (M.HpyAX) turned out to influence a large number of genes. Except for Type III MTase, M.HpyPIX, these are solitary Type II MTases. In contrast, the Type I system HpyAXIII (CTAN_8_TGT) and the Type III system HpyAXI (GCAG) have only minor influence. We could not detect any significant influence of the Type IIP RM system HpyPVIII (TCNNGA).

We found that the knockout of one MTase gene often affects expression of other MTases [Figures 1C**(ii–iv)**]. We extracted several elementary patterns in their interaction [Figure 1C**(iv)**]. One MTase’s expression may be stimulated positively by multiple (up to three) MTases. One MTase may have a positive regulator MTase and a negative regulator MTase [see also Figure 1D**(iii)**]. When one MTase positively regulates another MTase, a 3rd MTase may positively regulate both the MTases, negatively regulate both the MTases, or regulate the two MTases in the opposite directions. A few Type II MTase genes (5mC MTase targeting GCGC; 6mA MTase targeting GAAGG) were repeatedly detected as a regulatory target of other MTases. Presence of these interactions likely modify action of one MTase on the methylome, transcriptome and phenotype.

Expression of Type II systems, HpyPI, HpyPVII, or HpyAX, was not affected by knockouts of Type I or Type III methylation systems, but instead these Type II MTases affected expression of Type I, Type IIG, or Type III RM systems [Figures 1C**(ii,iii)**]. Because Type I, Type IIG and Type III RM systems change their TRDs over time (Figure 1B, “Introduction”), this apparent hierarchy between two groups of RM systems could be of some biological significance.

**FIGURE 3:**
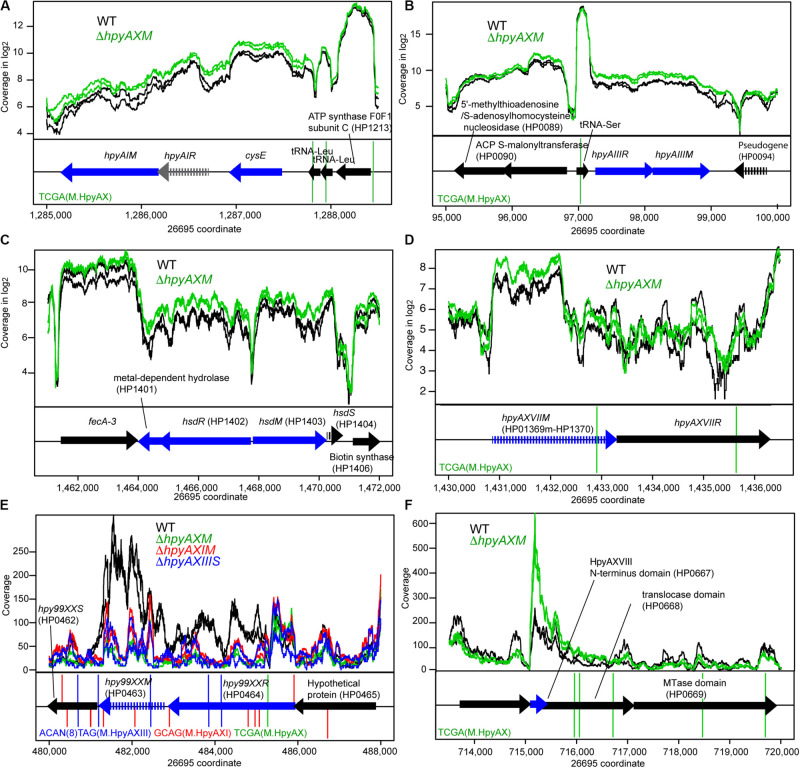
Effects of methyltransferase knockouts on transcript level of several methyltransferase loci. **(A)** HpyAI locus. **(B)** HpyAIII locus. **(C)** HP1403 locus. **(D)** HpyAXVII locus. **(E)** Hpy99XX homolog locus. **(F)** HpyAXVIII locus. The parental strain is 26695. Except for **(E,F)**, read coverage is shown in logarithmic scale. A blue arrow indicates a differentially expressed gene. A vertical line indicates a copy of methylation motif. A broken arrow indicates a pseudogene. The transcripts are from both strands as RNA-seq for strain 26695 was carried-out in “non-stranded” protocol.

**FIGURE 4:**
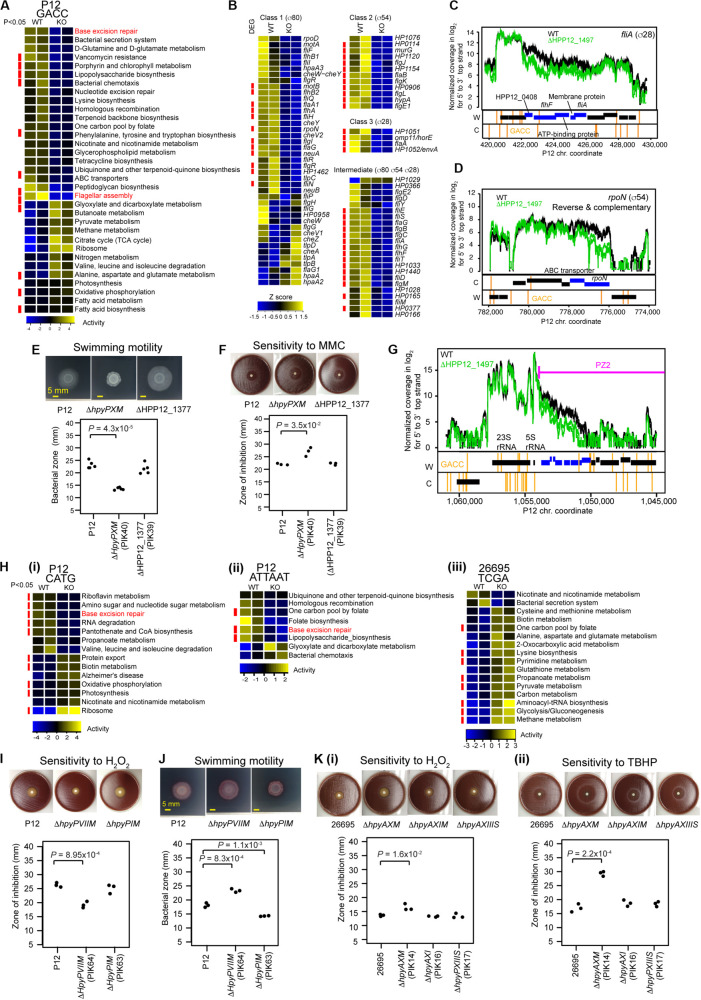
Transcriptome and phenotype changes in methyltransferase knockouts. **(A)** Transcriptome changes at the pathway level in Δ*hpyPXM*. Top 33 KEGG pathways with *P* < 0.1 (*n* = 2) in Welch’s *t*-test are shown. A pathway category with *P* < 0.05 is indicated by a red side bar. **(B)** Shut down of motility regulation in Δ*hpyPXM* revealed by transcriptome changes at the gene level. Read count was scaled to Z-score. A category of enrichment (*P* < 0.05) is indicated by a red side bar. **(C)** Read coverages in the parent and the knockout (upper panel) and GACC motif distribution (lower panels) around *fliA* (for sigma28). **(D)** Read coverages and GACC distribution around *rpoN* (for sigma54). A blue box indicates a differentially expressed gene. A vertical line indicates a copy of the methylation motif. **(E)** Swimming motility. Significance of the difference was evaluated by two-tailed Welch’s *t*-test. **(F)** Sensitivity to mitomycin C (MMC). **(G)** Transcriptome changes at the pathway level in Δ*hpyPIM* (CATG), in Δ*hpyPVIIM* (ATTAAT), and in Δ*hpyAXM* (TCGA). **(H)** Increased oxidative stress resistance in Δ*hpyPVIIM* (ATTAAT). **(I)** Increased swimming motility in Δ*hpyPVIIM* (ATTAAT). **(J)** Reduced oxidative stress resistance in Δ*hpyAXM* (TCGA). **(K) (i)** Hydrogen peroxide. **(ii)** *tert*-butyl hydrogen peroxide (TBHP). Statistical significance of the difference was evaluated by two-tailed Student’s *t*-test.

Some of the target MTase genes carry motif sequence copies of the controlling MTase in their coding or upstream regions (Figures 3ABD–F, Supplementary Figures S6A–D), which suggests gene expression control through local methylation. We also found that some MTases control expression of known transcription factors [Figures 1C**(I,iii)**]. Inactivation of HPP12_1365 reduced expression of its downstream genes encoding a two-component signal transduction system, which likely explains the transcriptome change. GACC motif is abundant in the windows covering PZ1, PZ3, and a 23S rRNA gene along the genome (Supplementary Table S2, Supplementary Figure S1) as we mentioned above. Upon inactivation of *hpyPXM*, reduced expression was observed downstream of a 23S rRNA gene including the adjacent PZ2 region (Figure 4G). Parts of genomic islands (PZ2, PZ3) with high GACC frequency reduced expression in *hpyPXM* knockout (Supplementary Figure S1). Activation of mobile DNA transcription by methylation is the opposite to what is commonly observed in eukaryotes (Slotkin and Martienssen, 2007).

To address whether the MTases contribute to adaptive phenotype, we first determined the effect of the knockouts on the growth (Figure 2D). We detected a difference for HpyPI (CATG), HpyPVII (ATTAAT), HpyPIX (GACC), HpyAX (TCGA), for which we also detected a large transcriptome change (Figure 2C). Among these, HpyPIX (GACC) knockout increased growth, whereas the other knockouts decreased (Figure 2D and Supplementary Figure S2).

### Gm6ACC MTase Has Positive Effect on Motility and DNA Damage Tolerance

HpyPIX (GACC, Type III) was previously shown to switch ON/OFF by simple-repeat length changes and affect expression of 6 genes (Srikhanta et al., 2011). From our transcriptome data, we evaluated its gene regulation activities at the pathway level using KEGG onthology. Top 33 categories changed are shown in Figure 4A. Among those, more than half categories decreased activity. These include pathways associated with DNA replication and repair (ko03410, base excision repair (Supplementary Figure S3A); ko03420, nucleotide excision repair; ko03440, homologous recombination) and cell motility [ko02030, bacterial chemotaxis; ko02040, flagellar assembly (Supplementary Figure S3D)].

The knockout showed a larger clear zone around mitomycin C, a DNA damaging agent, on blood agar (Figure 4F) (*t* = −4.8, df = 2.1, *P* = 0.035 in two-tailed Welch’s *t*-test). In the swimming motility assay on soft agar, the migration zone was significantly smaller in the knockout (Figure 4E) (*t* = 13.5, df = 4.9, *P* = 4.3 × 10^–5^, in two-tailed Welch’s *t*-test). These indicate that M.HpyPIX has a positive role in DNA damage resistance and in motility.

Eighty-one genes suggested to be involved in motility of *H. pylori* (Rust et al., 2008) are classified into four according to the sigma factor involved in their transcription (Figure 4B). Most genes constituting this network reduced expression in the knockout except for 6 chemotaxis-related proteins and others (Figure 4B). 11/12 class 2 genes and 4/4 class 3 were repressed. Differentially expressed genes were significantly overrepresented in these classes (*P* = 5.6 × 10^–3^ for class 3, *P* = 5.4 × 10^–6^ for class 2, and *P* = 1.5 × 10^–4^ for intermediate in one-tailed Fisher’s exact test). In the intermediate class, 15/23 genes were repressed with differentially expressed genes overrepresented (odds ratio = 5.2, *P* = 1.5 × 10^–4^ in one-tailed Fisher’s exact test). These responses are consistent with the strongly reduced expression of *rpoN* (σ54) for class 2 (1/3.5) and *fliA* (σ28) for class 3 (1/2.6). GACC motifs are present in the operon-like gene clusters containing *fliA* and *rpoN* (Figures 4C,D), so that their methylation may directly influence the expression. These results suggest that repression of *fliA* (for σ28) and *rpoN* (for σ54) expression is a major cause of the shutdown of the motility network by the M.HpyPIX knockout.

Eleven pathway categories increased transcripts in the M.HpyPIX knockout (Figure 4A; Supplementary Data Set S1). These belong to carbohydrate metabolism (ko00020, TCA cycle, 10 genes; ko00620, pyruvate metabolism), energy metabolism (ko00190, oxidative phosphorylation), and lipid metabolism (ko00061, fatty acid biosynthesis; ko00071, fatty acid degradation), and translation (ko03010, ribosome). Expression levels of most members in the TCA cycle and ribosome categories were increased (Supplementary Figures S3B,C). This may explain increased growth in the M.HpyPIX knockout [Figure 2D**(vii)**].

### ROS Tolerance Is Negatively Regulated by ATTAm6AT MTase but Positively by TCGm6A MTase

In the M.HpyPVII (ATTAAT) gene knockout, genes encoding antioxidant proteins, thioredoxin-dependent alkyl hydroperoxide reductase (HPP12_1554), NAD(P)H-flavin oxidoreductase (FrxA), and iron-dependent superoxide dismutase (HPP12_1031), were activated (Supplementary Data Set S1). The *pfr (= ftnA)* gene for ferritin, an iron (Fe^2+^ ion) storage protein, tolerating cation-mediated radical generation (Bereswill et al., 1998), is also activated. The knockout formed a smaller inhibition zone than the parent strain P12 around a filter with hydrogen-peroxide, which generates oxidative stress (*t* = 8.9, df = 4, *P* = 9.0 × 10^–4^ in two-tailed Student’s *t*-test) (Figure 4H). These results indicate that M.HpyPVII has a negative effect on ROS tolerance [Figure 1D**(ii)**].

In Δ*hpyAXM* (TCGA), genes for antioxidant proteins, catalase (HP0875), thioredoxin (HP0824), NAD (P) H-flavin oxidoreductase (FrxA), and cysteine synthesis-associated proteins (MetB, CysK), were repressed (Supplementary Data Set S1). Both catalase and cysteine can confer tolerance to oxidative stress. Furthermore, iron (Fe^2+^ ion) transporter gene *ceuE* (HP1162) was found moderately activated [*q*.value = 7.5E-04, *m*.value = 0.59 in differential gene expression analysis (see “Materials and Methods”)]. As expected, a larger inhibition zone was observed for two ROS inducing agents, hydrogenperoxide and *tert*-butyl hydrogen peroxide (TBHP), in the Δ*hpyAXM* strain [Figures 4K**(i,ii)**]. When the intact *hpyAXM* gene was restored, the inhibition zone was increased (Supplementary Figures S4A,B). These results indicate that M.HpyAX (TCGA) has a positive role in oxidative stress resistance [see also Figure 1D**(ii)**].

Expression of *flaA*, the flagellin gene, and several chemotaxis genes were increased in the M.HpyPVII (ATTAAT) knockout [Supplementary Data Set S1, Figure 4H**(ii)**]. The migration zone was larger (Figure 4I) (*t* = −9.034, df = 4, *P* = 8.3 × 10^–4^ in two-tailed Student’s *t*-test). When the intact *hpyPVIIM* gene was restored, a smaller migration zone was observed (*t* = −7.4, df = 4, *P* = 1.8 × 10^–3^ in two-tailed Student’s *t*-test) (Supplementary Figures S4C,D). These results indicate that M.HpyPVII negatively regulates motility [Figure 1D**(i)**].

### Cm6ATG at Translation Start Codon Affects Gene Expression

**FIGURE 5:**
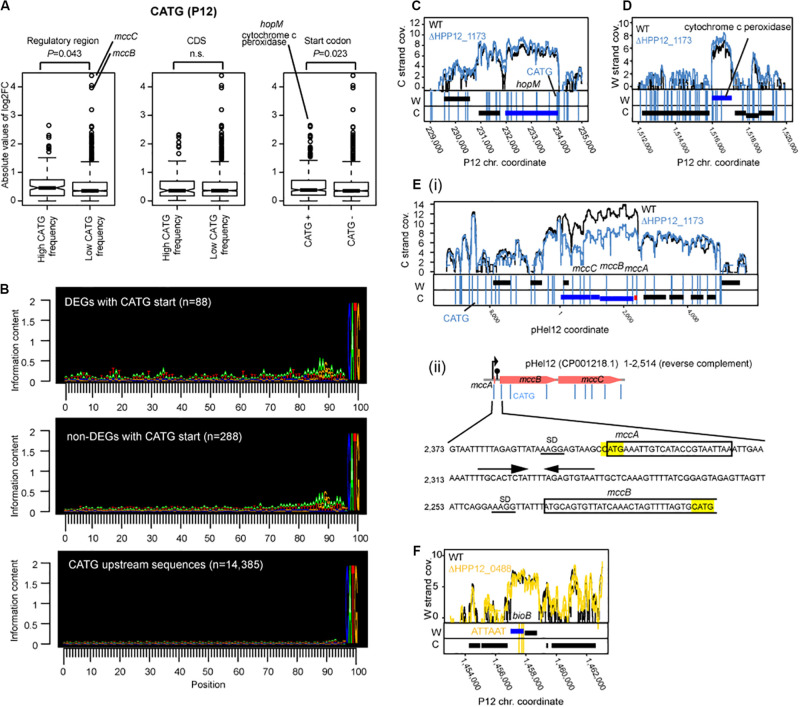
Effects of M.HpyPI (CATG) knockout on transcriptome. **(A)** Genes with high CATG motif abundance in the regulatory region or genes starting with CATG motif tend to be affected. Transcript abundance change was compared between two groups. The upstream 149 bp from the coding region was defined as the regulatory region. Statistical significance of the difference was evaluated by one-tailed Wilcoxon’s rank sum test; n.s.: *P* > 0.05. CDS, coding sequence. **(B)** Sequence conservation in the upstream region of CATG-starting genes. Conservation level was presented using Seqlogo (Schneider and Stephens, 1990). **(C)** Large transcript changes in *hopM*. **(D)** Large transcript changes in cytochrome c peroxidase gene. **(E) (i)** Large transcript changes in *mccB* and *mccC* genes for microcin on a plasmid. **(ii)** nucleotide sequence around *mccB* upstream region. CATG was highlighted in yellow. SD sequences are underlined. Putative terminator sequence (Zukher et al., 2014) were indicated by arrows. **(F)** Transcript abundance changes, upon M.HpyPVII knockout, in the *bioB* gene ending with three copies of ATTAAT. A blue box indicates a differentially expressed gene. A vertical line indicates a copy of a methylation motif.

To address whether methylation locally affects transcription as known for several solitary DNA MTases (Sánchez-Romero et al., 2015) and a Type I MTase (Furuta et al., 2014), we analyzed relation between occurrence of the methylation site and the transcript changes in detail for Cm6ATG (Δ*hpyPIM*), which includes a potential translation start codon ATG (Figure 5). When the group of genes with a CATG motif including start codon ATG and the group without one were compared, higher level of transcript changes by the knockout of Cm6ATG methyltransferase gene was detected in the former (*P* = 0.023, in one-tailed Wilcoxon’s rank sum test) (Figure 5A, right). Therefore, such start codon methylation somehow promotes transcription.

The transcript fold change was also significantly higher in genes with higher motif frequency in the upstream regulatory region (*P* = 0.043, one-tailed Wilcoxon’s rank sum test) (Figure 5A, left). We do not know whether this effect is due to the presence of an initiator peptide starting with CATG. There was no such effect detected for the CATG frequency within the coding sequence (Figure 5A, middle).

Upstream of the gene-starting CATG there are sequences characteristic of SD (Figure 5B, top panel and the middle panel as opposed to the bottom panel). When CDSs starting with CATG were separated into two groups: one carrying typical SD sequence “AAGG” within 15 bp from ATG (*n* = 207) and the other without “AAGG” (*N* = 176), the level of gene expression change (absolute value of *m*.value) was greater in the former group (*p*-value = 0.01429, one-tailed Wilcoxon rank sum test.

Further upstream, the differentially expressed genes with CATG start show more of the features of transcriptional promoters for housekeeping σ^80^, consisting of an extended Pribnow box and a periodic AT-rich signal (Sharma et al., 2010), than those not differentially expressed genes with CATG start (Figure 5B). These observations suggest that some association between start codon methylation and nearby upstream transcription initiation underlies the transcript changes in the knockout.

The largest transcript abundance difference by the knockout among the genes with CATG start were found for *hopM*, encoding an outer membrane protein, and for cytochrome c peroxidase, an electron transfer protein (Figure 5A right, CD). They both carry a cluster of CATG sites around the start codon (some in *dnaE*, for DNA polymerase III, neighboring the cytochrome c peroxidase gene).

Among the genes not starting with CATG, the largest transcript differences by the knockout was observed for microcin (a bacteriocin) genes, *mccB and mccC* [Figures 5A,E**(i,ii)**]. The *mccABC* operon carries a copy of CATG in the unstraslated region, the second CATG at the start codon for *mccA*, encoding a small peptide and not counted as a gene in our analysis, and the third CATG in the 5′ side within *mccB* gene. Palindromic DNA sequences potentially forming a stem-loop structure on RNA leading transcription termination is found in this region [Figure 5E**(ii)**]. There was no CATG in the corresponding region in the *E. coli mccABC* operon. Ribosome-controlled transcription termination is involved in translation of these genes (Zukher et al., 2014). These again suggest some association between methylation, transcription, and translation.

For Δ*hpyPAX* (TCGA), Δ*hpyPVIIM* (ATTAAT) and Δ*hpyPXM* (GACC), no such difference between the groups was detected (Supplementary Figure S5). The highest transcript fold changes were observed in ‘inactivated’ MTase genes: GANTC-methylating MTase for Δ*hpyPVIIM* (Supplementary Figure S5B) and a Type I M gene (HP0463) for Δ*hpyPAX* (Supplementary Figure S5). This again indicates MTase-mediated control of expression of another MTase. Auto-repression activity may be present in these target MTases in their intact forms as in some MTases (Karyagina et al., 1997; Mruk et al., 2011) and the gene inactivation may have led to loss of such auto-repression and to the large effects.

Because ATTAAT, the methylation motif of HpyPVIIM, contains TAA, a translation stop codon, we sought for genes with this stop codon among the differentially expressed genes between Δ*hpyPVIIM* (ATTAAT) and its parent. We noticed one such case involving *bioB* (Figure 5F, Supplementary Data Set S1). This gene carries three copies of ATTAAT toward its 3′ end and the last copy was annotated as the stop codon.

## Discussion

These results revealed a complex gene expression regulation network involving many DNA methyltransferases, as well as known transcription regulators, as hub regulators (Figure 1C). The MTases regulate each other in several patterns so that methylation activity of one MTase influences various aspects of cell phenotypes likely increasing phenotypic variation, consistent with the epigenome-base adaptive evolution model. Many of these MTase hubs are present in only limited lineages (Figure 1B) and/or change their sequence specificity (see section “Introduction”), frequently remodeling the network. The system may evolve irreversibly through these changes. Such irreversibility distinguishes the system from the known reversible phenomena involving simple-repeat length changes or site-specific recombination (Sánchez-Romero et al., 2015). The network may be worth the name of *Firework*. Genome comparison revealed various ways in which the DNA methyltransferases change their sequence specificity in *H. pylori*: (1) amino-acid substitution (Furuta et al., 2014), (2) homologous recombination moving target recognition domains (Kobayashi, 2012a, 2013; Furuta et al., 2014), and (3) changes in the number of central repeats in the S subunit of Type I system (Price et al., 1989; Andres et al., 2010). Although (2) and (3) appear more rapid than (1), there has been no attempt to measure these rates in the microevolution process or in the laboratory setting.

The Firework metamorphoses likely in search for adaptation. The phenotype affected by MTases includes motility, which is central to *H. pylori*’s establishment and persistence of infection. Two MTases have opposing effects on motility, an accelerator and a brake [Figure 1D**(i)**], likely for fine tuning. ROS from the host immune system damages DNA and proteins of *H. pylori* but also acts as a signal controlling redox status of enzymes and regulatory proteins (Ortiz de Orué Lucana et al., 2012). Two MTases are involved in ROS metabolism in opposite ways [Figure 1D**(ii)**]. Their overlap in some physiological functions may allow loss of one of the MTase genes during long-term *H. pylori* evolution. Even in a small *H. pylori* population in Japan, we observed sporadic loss of *hpyAXM*-equivalent gene (Kojima et al., 2016). Genes/apparent operons detected as differentially express genes in more than three MTase knockouts include *groEL* chaperone operon (4 knockouts, 3 activation, 1 repression), *dnaK* for a chaperon, *vacA*, HPP12_1177 (F0F1 ATP synthase subunit), HPP12_0834 (RNA-binding protein), *mdaB* (antioxidant protein), *flaA* (flagellin), *ureB* (urease), HPP12_0571 (membrane protein), *babB* (outer membrane protein), and *mcc* (microcin) operon on the plasmid pHPP12 (Supplementary Data Set S1). Some of these genes are involved in host interaction and virulence (*vacA, flaA*, *ureB*, *babB*, *ureB*) [Rust et al., 2008; Figures 1E**(i,ii)**] and in bacterial interaction (*mcc*) (Zukher et al., 2014). Effect of MTase on chaperon expression was reported earlier (Srikhanta et al., 2005; Vitoriano et al., 2013). The base-excision repair category (KEGG id 03410) is repressed in three MTase knockouts (Figures 4A,H; Supplementary Figure S3A). We do not know whether this is related to the presence of a base-excision type restriction enzyme in *Helicobacter* (Miyazono et al., 2014; Kojima and Kobayashi, 2015). These observations imply that the chaperones, the DNA repair systems, the motility system, the redox system, and the host interaction systems can sense the activity of MTases.

Reversible changes of gene expression, phase variation, may result from ON/OFF of DNA methyltransferase genes by simple-repeat length changes (Srikhanta et al., 2009) and from generation of a specificity subunit by site-specific recombination events in a restriction-modification system (Manso et al., 2014; Li et al., 2016). The unique features of the present system are that (1) DNA methyltransferases form a hierarchical network, (2) the specificity changes can take place in many of the hub DNA methyltransferases by movement of the domains (in addition to ON/OFF of the DNA methyltransferase genes) (3) and the overall change in the gene regulation network appears irreversible.

MTases regulate its expression and activity in various ways (Mruk and Kobayashi, 2014). One is auto-repression, which may explain increased expression of MTase genes (above). One MTase of *H. pylori*, M.HpyAXI (GCAG), changes its biochemical activity depending on pH (Banerjee and Rao, 2011). M.EcoRI transcript overlaps with an antisense RNA forming a bi-stable switch (Mruk et al., 2011). If an MTase can sense environmental signal, the Firework can be a simple system to generate phenotypic but heritable variations in a short time *in vivo*. To fully explore the potential for such “Lamarckian” evolution, further investigation is necessary on changes of genes and expression of MTases in response to environmental signals.

To our surprise, association of DNA methylation and translation initiation/termination in determining transcript abundance was suggested from multiple lines of observation with CATG, including a potential start codon ATG, and ATTAAT, including a stop codon TAA.

Our following observations suggest relation of DNA methylation at CATG at start codon and transcript abundance.

1. The genes with CATG start showed a higher level of transcript changes by the knockout of Cm6ATG methyltransferase gene (Figure 5A, right).
2. When CDSs starting with CATG were separated into two groups: one with SD and the other without one, the level of gene expression change was greater in the former.
3. The differentially expressed genes with CATG start show more of the features of transcriptional promoters (Figure 5B).
4. The CATG-starting genes with the largest transcript differences by the knockout (*hopM* and cytochrome c peroxidase) also carried CATGs around the start codon ATG (Figures 5C,D).
5. A large transcript differences by the knockout was observed for *mccBC* operon (Figures 5A,E), which carries a CATG in the upstream untranslated region and another CATG at the start codon for *mccA*. Ribosome-controlled transcription termination is involved in translation of these genes (Zukher et al., 2014).

These observations suggest that methylation at or around start codon affects transcription. We also found a case (Figures 5F, Supplementary Data Set S1), where methylation at three copies of ATTAAT at 3′ end (with the last copy annotated as the stop codon) decreases transcript. This suggests methylation at a potential translation stop codon may affect transcription.

**FIGURE 6:**
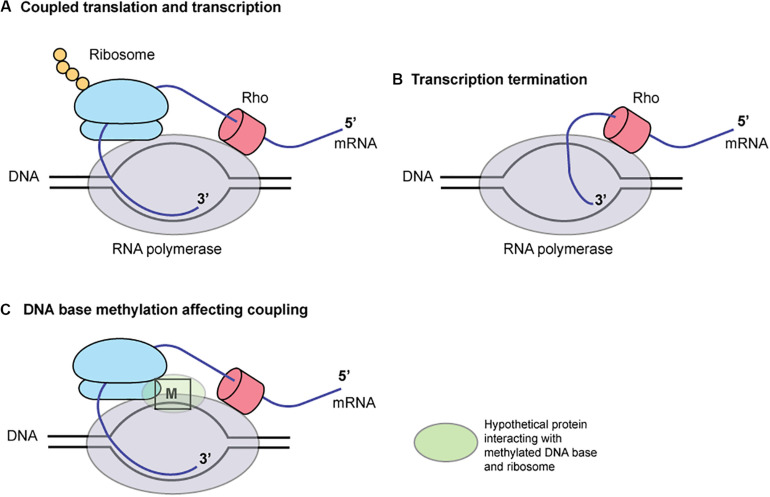
A hypothesis for the role of DNA base methylation in transcription-translation coupling. Translation by ribosome **(A)** prevents mRNA release by Rho **(B)**. A hypothetical protein binding the methylated base, **(C)** RNA polymerase and ribosome affects the coupling. M, a methylated base in DNA.

These findings may be explained by the coupling of translation and transcription in bacteria (Figure 6; McGary and Nudler, 2013). When coupled to RNA polymerase (RNAP), the translating ribosome ensures transcriptional processivity by preventing RNAP backtracking. The trailing ribosome “pushes” RNAP, thereby modulating the rate of transcription by creating a synchrony of mRNA production and protein synthesis. The first 50 nucleotides of transcribed mRNA are particularly prone to stalling and backtracking. The rate of translation depends on the mRNA secondary structures. The ribosome behaves as an mRNA helicase, disrupting mRNA duplexes. In a Cryo-EM structure of RNA polymerase – ribosome 30S subunit complex (Demo et al., 2017), the RNA exit tunnel of RNA polymerase aligns with the SD-binding site of 30S subunit. Ribosomal protein S1 forms a wall of the tunnel between RNAP and the 30S subunit, consistent with its role in directing mRNAs onto the ribosome. NusG, present in *E. coli* and *H. pylori*, can bind both RNA polymerase and ribosome to mediate the coupling (Krupp et al., 2019; Washburn et al., 2019). Some of these elements are close to the template DNA (Krupp et al., 2019) and may well be affected by DNA base methylation and proteins bound to the methylated DNA. Indeed, a palindromic DNA sequence generating a stem-loop structure on mRNA that might lead to transcript release by Rho are found between *mccA* and *mccB*. Type I methylation on a long palindrome decreases transcript in an operon-like structure (Furuta et al., 2014). Further informatic and experimental works are necessary to test this hypothesis.

While we were preparing this manuscript, one paper appeared (Srikhanta et al., 2017), which knocked out M.HpyPIX in P12 and confirmed its methylation motif as GACC and reported its effects on transcription and motility. One recent paper (Kumar et al., 2018) demonstrated effects of Tm4CTTC MTase on transcription, natural transformation and host interaction. Another paper (Estibariz et al., 2019) demonstrated that Gm5CGC methylation in the promoter affects transcription.

## Data Availability Statement

The datasets generated for this study can be found in the DDBJ Sequence Read Archive (https://www.ddbj.nig.ac.jp/dra/index-e.html) under accession numbers DRA005953, DRA004356, DRA003551, and DRA003688.

## Author Contributions

HY and IK conceptualized the study. HY, MF, YF, TS, TN, and SSh contributed to the formal analysis. HY, ER, MA, and TS investigated the study. YS, SSu, MH, AT, KS, and IK provided the resources. HY, MA, TS, and IK wrote the original draft. HY, MF, and IK reviewed, and edited the manuscript. IK supervised the study. All authors contributed to the article and approved the submitted version.

## Conflict of Interest

The authors declare that the research was conducted in the absence of any commercial or financial relationships that could be construed as a potential conflict of interest.

## Supplementary Material

FIGURE S1 Gene expression changes in high GACC frequency regions in the *hypoPXM* knockout.

FIGURE S2 Growth pattern of P12 methyltransferase gene knockouts.

FIGURE S3 Expression changes of genes in specific KEGG categories.

FIGURE S4 Gene restoration for Δ*hpyAXM* and Δ*hpyPVIIM*.

FIGURE S5 Effect of methylation in the regulatory and CDS regions on the transcriptome.

FIGURE S6 Transcript levels of the representative R-M systems under the control of multiple R-M systems in strain P12.

TABLE S1 Methylomes of methyltransferase knockout mutants.

TABLE S2 Genomic segments with high and low GACC abundance.

TABLE S3 Bacterial strains and plasmids used.

TABLE S4 Oligonucleotides used.

TABLE S5 qPCR for RNA-seq validation.

DATASET S1 Differential gene expression analysis.
