# Corrigendum: Networking and Specificity-Changing DNA Methyltransferases in *Helicobacter pylori*

**DOI:** 10.3389/fmicb.2020.596598

**Published:** 2020-09-22

**Authors:** Hirokazu Yano, Md. Zobaidul Alam, Emiko Rimbara, Tomoko F. Shibata, Masaki Fukuyo, Yoshikazu Furuta, Tomoaki Nishiyama, Shuji Shigenobu, Mitsuyasu Hasebe, Atsushi Toyoda, Yutaka Suzuki, Sumio Sugano, Keigo Shibayama, Ichizo Kobayashi

**Affiliations:** ^1^Department of Computational Biology and Medical Sciences, Graduate School of Frontier Sciences, The University of Tokyo, Tokyo, Japan; ^2^Institute of Medical Science, The University of Tokyo, Tokyo, Japan; ^3^Department of Bacteriology II, National Institute of Infectious Diseases (NIID), Musashimurayama, Japan; ^4^National Institute for Basic Biology (NIBB), Okazaki, Japan; ^5^School of Medicine, Chiba University, Chiba, Japan; ^6^Advanced Science Research Center, Kanazawa University, Kanazawa, Japan; ^7^Department of Basic Biology, School of Life Sciences, SOKENDAI (The Graduate University for Advanced Studies), Okazaki, Japan; ^8^Advanced Genomics Center, National Institute of Genetics, Mishima, Japan; ^9^Department of Infectious Diseases, School of Medicine, Kyorin University, Mitaka, Japan; ^10^Institut de Biologie Intégrative de la Cellule (I2BC), Université Paris-Saclay, Gif-sur-Yvette, France; ^11^Research Center for Micro-Nano Technology, Hosei University, Koganei, Japan

**Keywords:** epigenetics, methylome, DNA methylation, gastric cancer, epigenome, DNA methyltransferase, SMRT, PacBio

In the original article, there was an error. “HpyPIX” was misspelled as “HpyPX” throughout the article. The correct spelling is “HpyPIX”.

The authors apologize for this error and state that this does not change the scientific conclusions of the article in any way. The original article has been updated.

