# Supplementary material for: Networking and Specificity-Changing DNA Methyltransferases in Helicobacter pylori
Source: Front Microbiol. 2020 Jul 17;11:1628. doi: 10.3389/fmicb.2020.01628 (PMC7379913; doi:10.3389/fmicb.2020.01628)
Supplement: FIGURE S1 — Gene expression changes in high GACC frequency regions in the hypoPXM knockout. [file Presentation_1.pdf]

Supplementary Figures

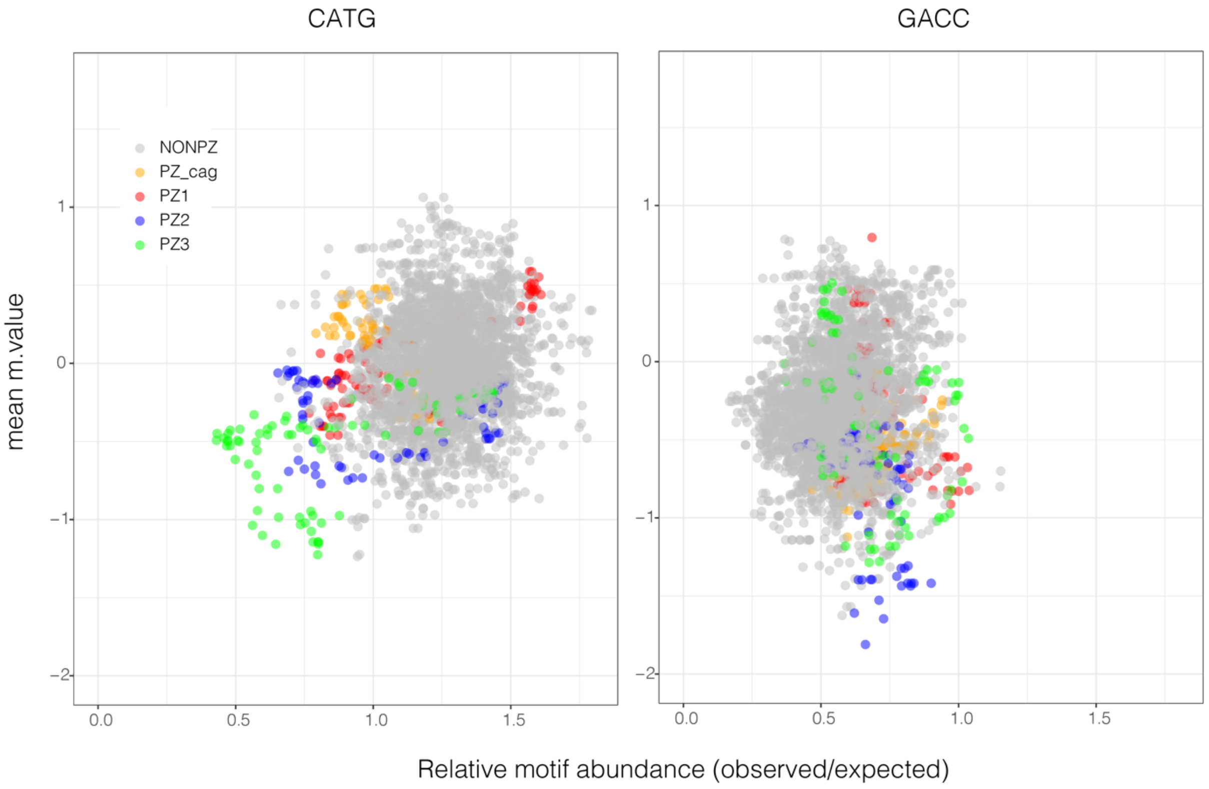

Supplementary Figure S1. Gene expression changes in high GACC frequency regions in the *hpyPXM* knockout. Y-axis indicates mean of gene expression changes in each 10 kb sliding window. X-axis indicates the relative motif abundance (observed /expected)

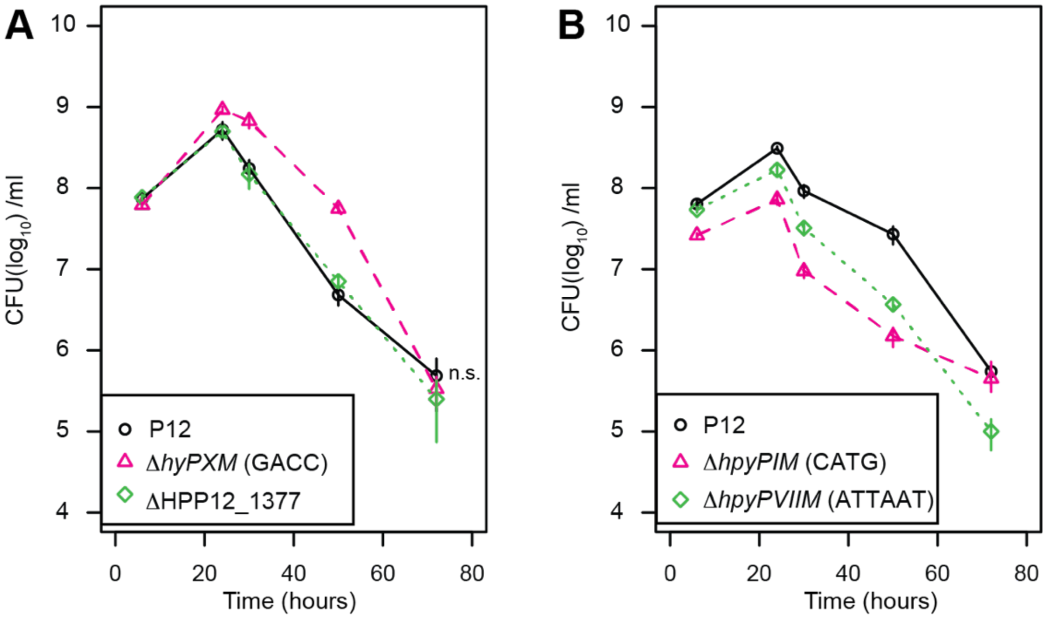

Supplementary Figure S2. Growth pattern of P12 methyltransferase gene knockouts. (**A**) Colony forming unit changes in the parallelly grown cultures of P12, Δ*hpyPXM*, and Δ*HPP12_1377.* N=4. **(B)** Colony forming unit changes in the parallelly grown cultures of P12, Δ*hpyPIM*, and Δ*hpyPVIIM*. N=4. Error bar represents standard error of the mean.

(A) Base-excision repair

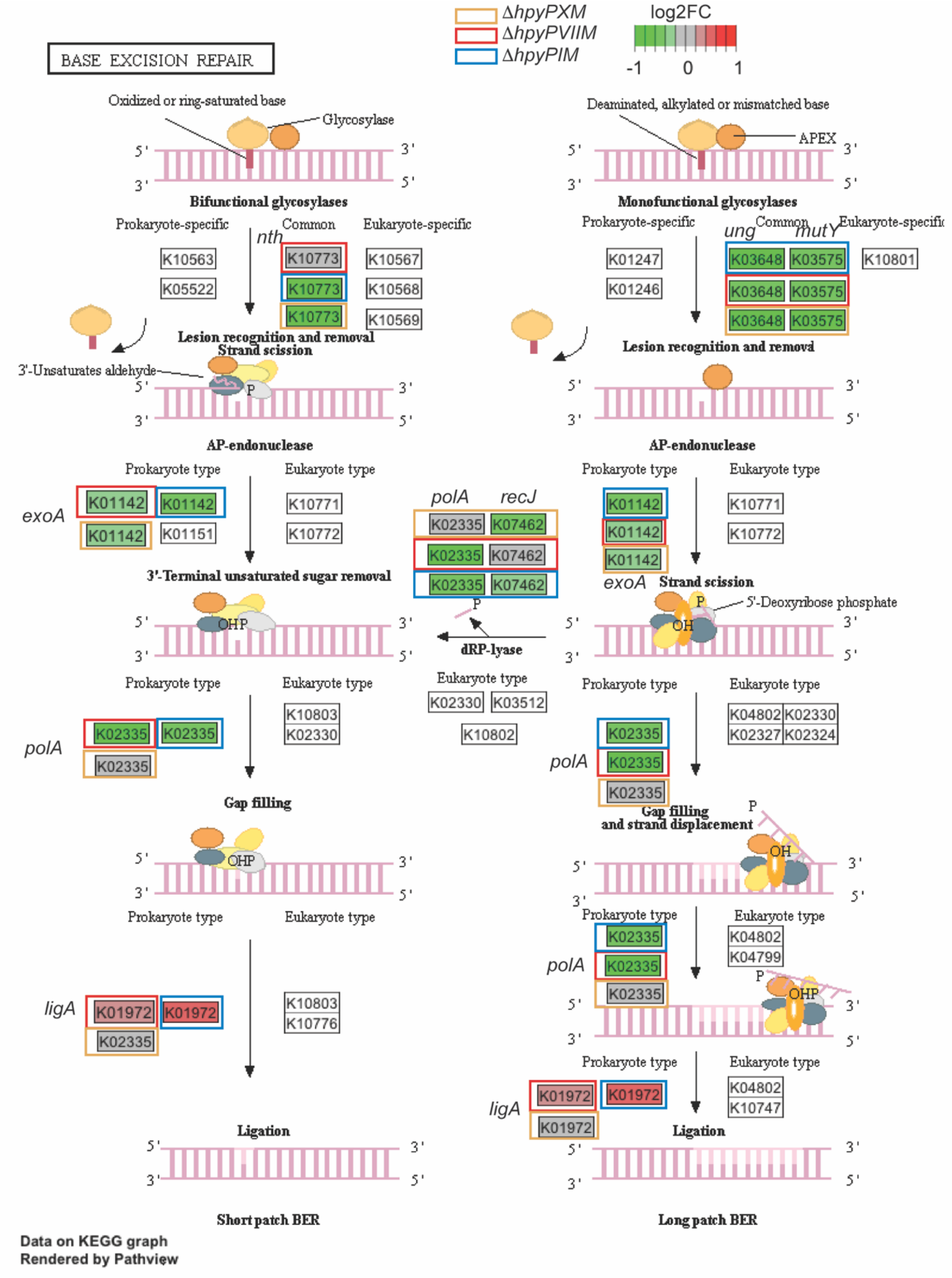

(B) TCA cycle (Δ*hpyPXM*)

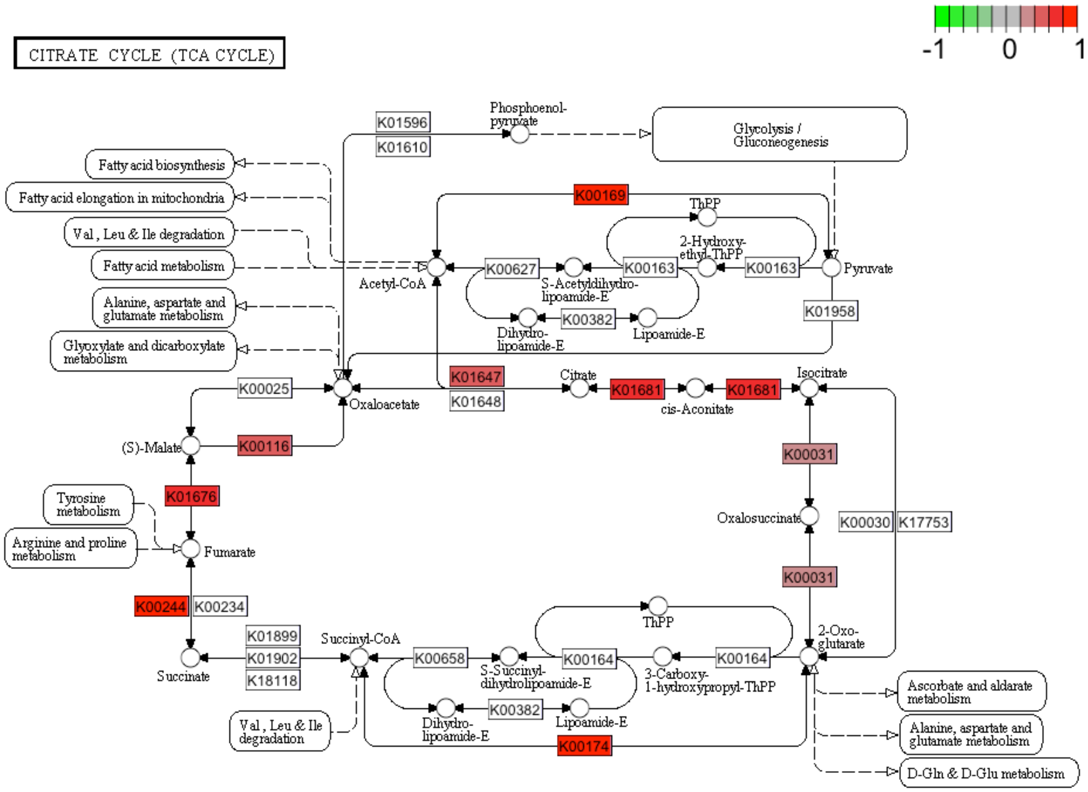

(C) Ribosome (Δ*hpyPXM*)

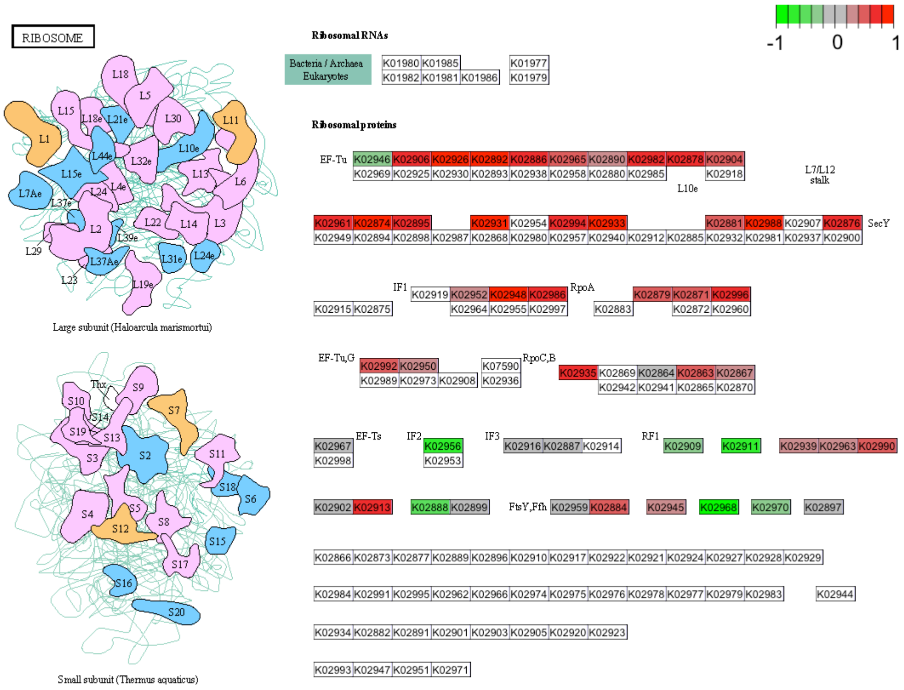

(D) Flagellar Assembly (Δ*hpyPXM*)

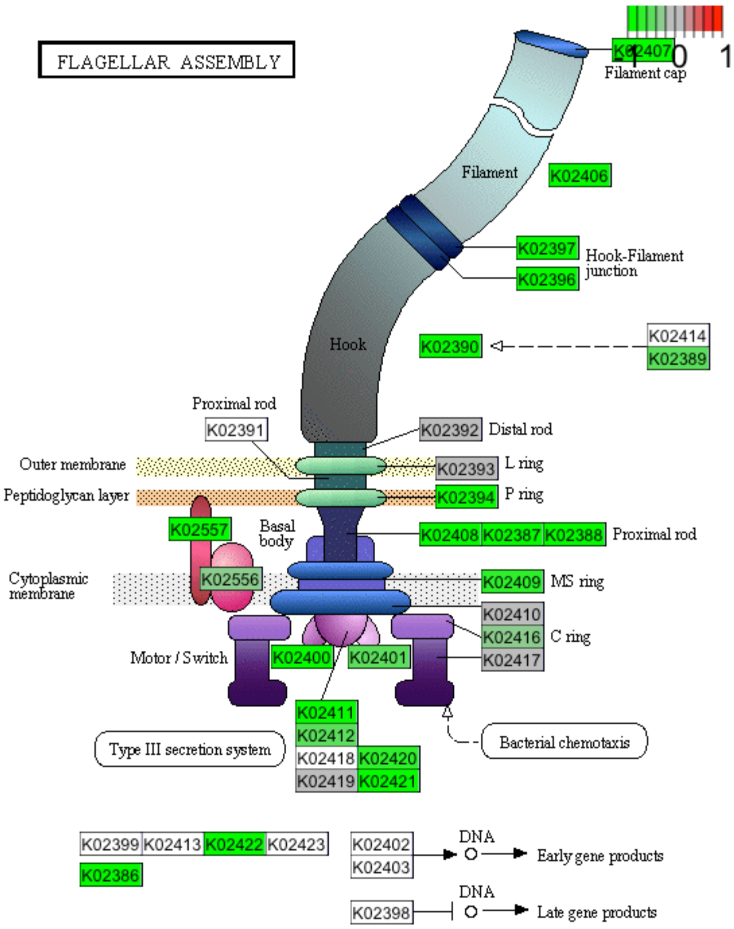

Supplementary Figure S3. Expression changes of genes in specific KEGG categories. **(A)** Base-Excision-Repair category in Δ*hpyPIM* (CATG), Δ*hpyPXM* (GACC) and Δ*hpyPVIIM* (ATTAAT). **(B)** Ribosome in Δ*hpyPXM*. **(C)** TCA cycle in Δ*hpyPXM*. **(D)** Flagellar assembly in Δ*hpyPXM*. Color code represents log2FC: $\log_2$[fold change]. Pathview representation.

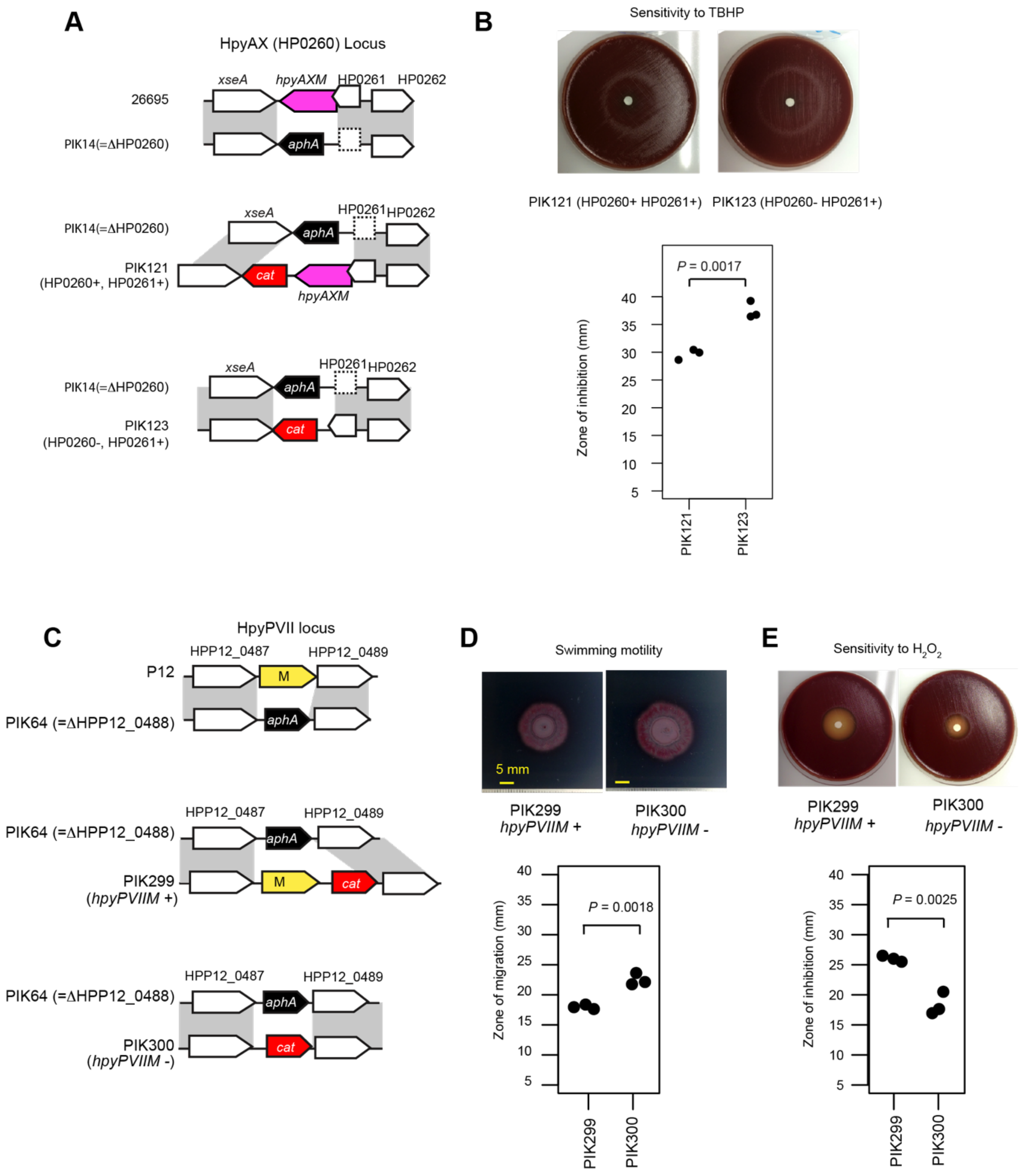

Supplementary Figure S4. Gene restoration for Δ*hpyAXM* and Δ*hpyPVIIM* indicates that the *hpyAXM* (= HP0260) gene increases oxidative stress resistance. (A) Change in HpyPAX locus. In the initial knockout strain, a part of the upstream gene (HP0261) was deleted. We constructed two isogenic strains: one with the intact HP0261-HP0260 (*hpyAXM*) region and the other with the intact HP0261 but without *hpyAXM*. (B) Oxidative stress resistance analysis. TBHP: *tert*-butyl hydroperoxide. (C) Changes in the HpyPVII locus. (D) Swimming motility assay. (E) Oxidative stress resistance analysis. Statistical significance for the difference was evaluated by Weltch's t-test.

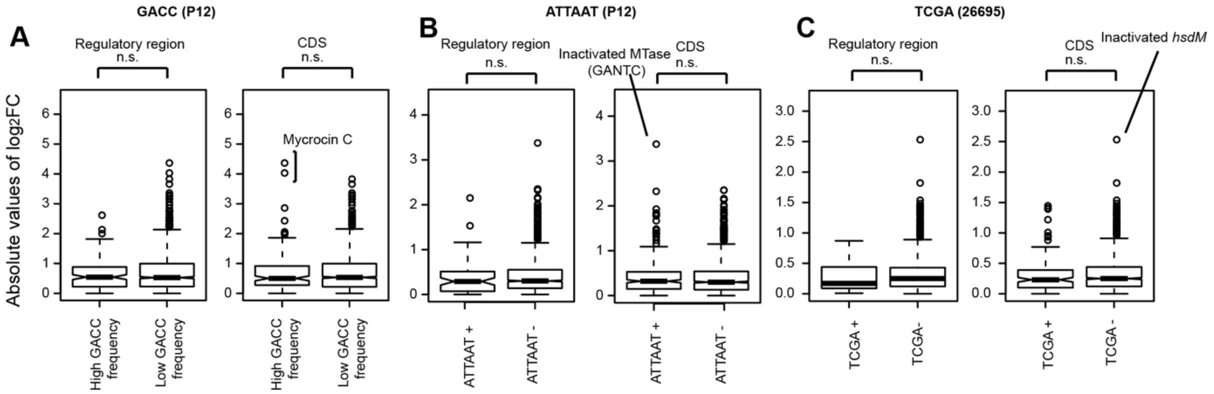

Supplementary Figure S5. Effect of methylation in the regulatory and CDS regions on the transcriptome. (A) HpyPXM (GACC) knockout. (B) HpyPVIIM (ATTAAT) knockout. (C) HpyAXM (ATTAAT) knockout. Gens were classified into two groups: Each gene was classified into two categories: with and without a motif for less frequently occurring motifs, TCGA and ATTAAT; high and low motif frequencies for frequently occurring motifs, GACC. No significant difference was observed between the two groups in these transcriptome data sets. $P > 0.05$ in Wilcoxon's rank sum test.

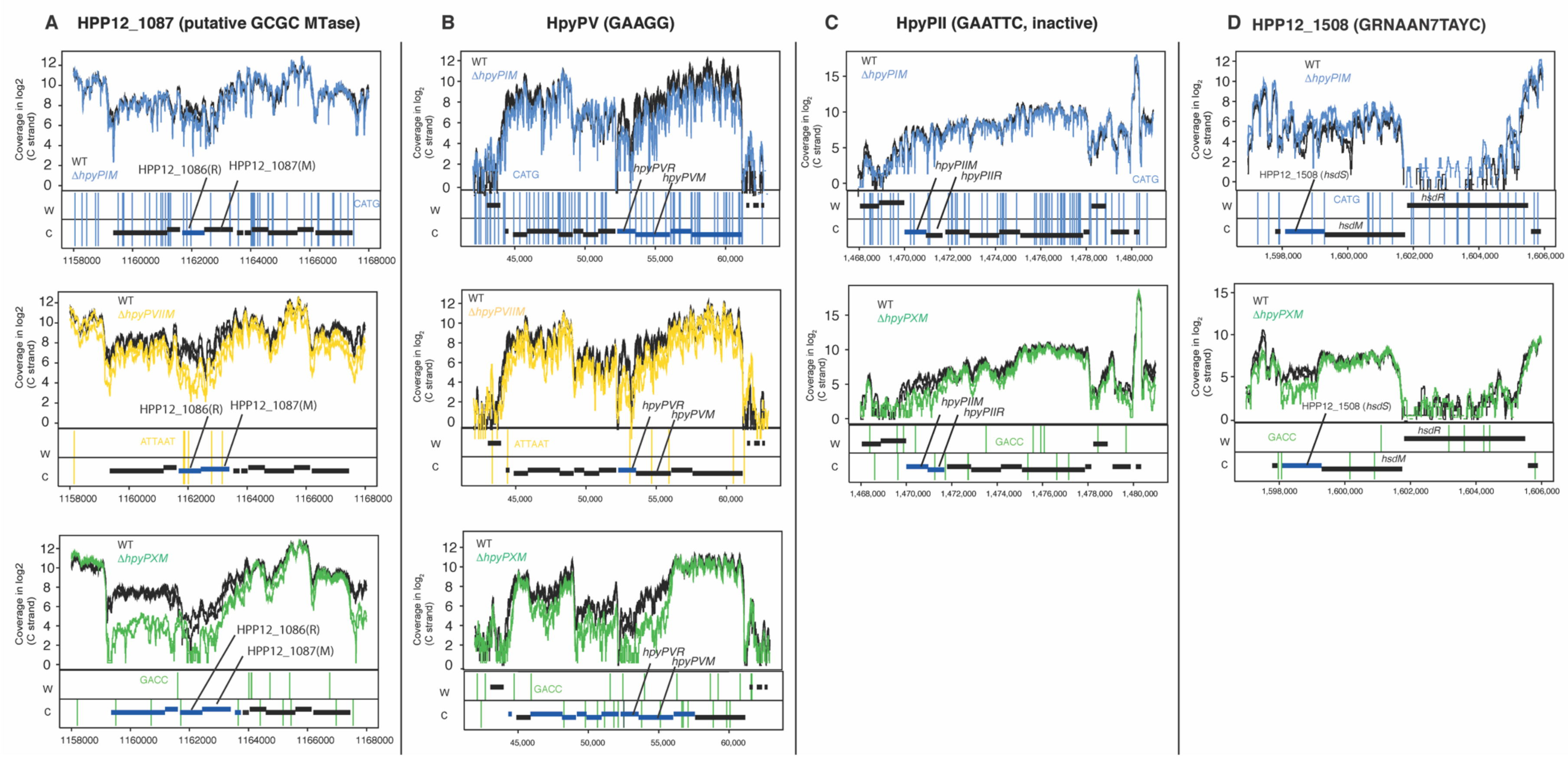

Supplementary Figure S6. Transcript levels of the representative R-M systems under the control of multiple R-M systems in strain P12. (A). HPP12_1087 locus, (B) HpyPV locus, (C), inactive HpyPII locus, (D) HPP12_1508 locus. Each plot indicates changes in read coverage in the C strand. Horizontal bar below the coverage plot indicates coding region. Differentially expressed genes are shown in blue. Vertical bar in the lower boxes denotes methylation position of the indicated motif.
